# Metallic Combinations of Muriatic Acid, in Its Different States
*We are favoured by Mr. Chenevix with his Observations and Experiments upon oxygenized and hyperoxygenized Muriatic Acid, and upon some combinations of the Muriatic Acid in its three states; which have been read at the Royal Society, and we have great pleasure in selecting these experiments as highly interesting to chemists and practitioners in general. Edit.


**Published:** 1802-06

**Authors:** R. Chenevix


### *Metallic Combinations of Muriatic Acid, in its different States.** By R. CHENEVIX, Eſq. F. R. S. R. M. I. A.

THE action of hyperoxygenized muriatic acid upon metals, is, as may well be expected, rapid, and without diſengagement of gas. It appears to diſſolve every metal, not excepting gold and platina. If the metal be preſented to the acid at the moment when it is diſengaged from the ſalt, inflammation enſues; and the phenomena of light and heat vary according to the metal; but the ſalts thus produced are merely muriates. In order to form real hyperoxygenized muriates, it is neceſſary to take the

* We are favoured by Mr. Chenevix with his Obſervations and Experiments upon oxygenized and hyperoxygenized Muriatic Acid, and upon ſome combinations of the Muriatic Acid in its three ſtates; which have been read at the Royal Society, and we have great pleaſure in ſelecting theſe experiments as highly intereſting to chemiſts and practitioners in general. EDIT.

the metal in its fulleſt ſtate of oxidizement, and combine it with the acid, either by double decompoſition, or by paſſing a current of oxygenized muriatic acid gas through the oxide ſuſpended in water. The acid is thus ſeparated into muriatic and hyperoxygenized muriatic acid; and, in theſe ſtates, combines with the metallic oxide. The metallic hyperoxygenized muriates are different, in every reſpect, from the metallic muriates. Red oxide of iron is diſſolved with difficulty. Oxide of copper more eaſily. Red oxide of lead exhibits the ſame appearances, during its combination with this acid, as with nitric acid. When nitric acid is poured, even in exceſs, upon red oxide of lead, only a part of the oxide is diſſolved, unleſs heat be applied; and what remains becomes a blackiſh brown powder. But if metallic lead be added, in a juſt proportion, all the red oxide diſappears, and none of the brown powder is formed; neither is there any diſengagement of nitrous gas when the metallic lead is diſſolved. The precipitates cauſed in either caſe, by pouring an alkali into the nitric ſolution, are yellow. Hence it appears, that red oxide of lead contains too much oxygen to be diſſolved by nitric acid. One part of the oxide takes up the exceſs of oxygen, and becomes brown; while the portion which loſes oxygen, becomes yellow, and is ſoluble in nitric acid. The preſence of metallic lead promotes the total ſolution of the red oxide, by taking up the ſuperabundant oxygen. I found that a current of oxygenized muriatic acid gas, like the nitric acid, diſſolved a part of the red oxide, and cauſed the brown powder to be formed, upon which it could not act. Hyperoxygenized muriate of lead is much more ſoluble than muriate of lead; and the acid is very ſlightly attracted by the baſis.

But, of all the metallic ſalts formed by the combination of the muriatic acid, in any of its different ſtates, none ſo much deſerve attention as thoſe which have for their baſes the oxides of mercury. The nature of the ſalts which reſult from the combination of common muriatic acid with the different oxides of this metal, has been ſtated in the moſt contradictory manner by different chemiſts. But as the knowledge of hyperoxygenized muriatic acid has thrown ſome light upon the true ſtate of calomel and corroſive ſublimate,* I muſt beg leave to dwell at

---

* I regret very much, that I am under the neceſſity of uſing theſe unmeaning terms. But the French Nomenclature has made no diſtinction between ſalts formed by metallic oxides in different ſtates of oxidizement, except by the colour, which is an extremely defective and unmeaning method. At all events, this metal is ſo uncomplaiſant as to retain the white colour, in its different oxides combined with muriatic acid. I prefer, however, uſing the old name, to propoſing any proviſional ſubſtitute that might be found defective.

at ſome length upon this important part of my ſubject. It would be uſeleſs to repeat the opinions of the old authors, who have treated of corroſive ſublimate, and of calomel. They are to be found in the works of thoſe reſpective chemiſts, and I muſt refer to them for particulars.

In the Memoirs of the Academy of Sciences of Paris, for 1780, we find a paper of Mr. Berthollet, upon the cauſticity of metallic ſalts; in which he appears to think, that the acid in corroſive ſublimate is in the ſtate of what was then called dephlogiſticated marine acid. In 1785, when he had examined the oxygenized muriatic acid with more care, he renounced his former opinion; and gave the reaſons why he no longer adhered to it. Some late experiments of Mr. Prouſt ſhew, that this chemiſt thinks as Mr. Berthollet now does. And theſe may be ranked among the firſt of modern authorities.

Notwithſtanding thoſe opinions, Mr. Fourcroy, in his Syſteme des Connoiſſances Chimiques, ſtill conſiders corroſive ſublimate as a hyperoxygenized muriate of mercury, and deſigns it throughout by that name. This chemiſt, one of the founders of the methodical Nomenclature, is too well acquainted with its principles, to apply the term hyperoxygenized muriate to any thing but a combination of hyperoxygenized muriatic acid. It is evident, therefore, that he conſiders the portion of oxygen, which, in equal quantities of corroſive ſublimate and calomel, is greater in the former, to be combined with the acid, and not with the oxide of mercury. As ſoon as I have ſtated ſome experiments that prove Mr. Fourcroy's opinion to be erroneous, and endeavoured to eſtabliſh the analyſis of corroſive ſublimate and of calomel, I ſhall take notice of a ſalt hitherto unknown, which really is hyperoxygenized muriate of mercury.

I took a portion of corroſive ſublimate, and precipitated by potaſh. The liquor was filtered; and, upon being tried, nothing but muriate of potaſh was found. No reagent could diſcover the ſmalleſt trace of hyperoxygenized muriatic acid.

Sulphuric, nitric, phoſphoric, and many other acids, poured upon corroſive ſublimate, did not diſengage either muriatic or hyperoxygenized muriatic acid. Nitrate of ſilver, poured into a ſolution of corroſive ſublimate, gave an abundant white precipitate.

From theſe experiments it is evident, that muriatic acid, not hyperoxygenized muriatic acid, is combined with the oxide of mercury in corroſive ſublimate.

To determine the proportions of this ſalt, I took one hundred parts, and precipitated by nitrate of ſilver. I then took another hundred, and precipitated by potaſh. The reſult of theſe theſe two experiments was ſuch as to eſtabliſh the proportions of corroſive ſublimate as follows:

| | |
|---|---|
| Oxide of mercury — — — — | 82 |
| Muriatic acid — — — — — | 18 |
| | 100. |

But the acid of this ſalt not being charged with a ſuperabundance of oxygen, we muſt look for the exceſs in the metallic oxide. I took 100 grains of mercury, and diſſolved them in nitric acid; then poured in muriatic acid; and, at a very gentle heat, evaporated to dryneſs. I afterwards ſublimed, in a Florence flaſk, the ſalt that remained, and obtained 143,5 of corroſive ſublimate. But 143,5 of corroſive ſublimate contain 26 of acid, which will leave 117,5 for the mercurial oxide; and, if 117,5 contain 100 of mercury, 100 of the oxide will contain 85. Therefore, the oxide of mercury, in corroſive ſublimate, is oxidized at the rate of 15 per cent.

To determine the proportions in calomel, I diſſolved 100 grains of it in nitric acid. The phenomena of the ſolution have been ſo accurately deſcribed by Mr. Berthollet, that I ſhall not repeat them. I precipitated by nitrate of ſilver, and obtained a quantity of muriate of ſilver, correſponding with 11,5 of muriatic acid. The oxide of mercury I obtained apart. Therefore, calomel is compoſed of,

| | |
|---|---|
| Oxide of mercury — — — | 88,5 |
| Muriatic acid — — — — | 11,5 |
| | 100,0. |

To aſcertain the ſtate of oxidizement of the oxide in calomel, I took 100 grains, and boiled them with nitro-muriatic acid; then evaporated very ſlowly, and ſublimed as above. The calomel was totally converted into corroſive ſublimate, and weighed 113. But 113 of corroſive ſublimate contain 20,3 of muriatic acid, of which, 11,5 were originally in the calomel. The total addition of weight was 13. But the quantity of acid in theſe 13, amounts to 20,3 — 11,5 = 8,8. Therefore, 13—8,8 = 4,2, remain for that part of the additional weight which is oxygen. On the other hand, 100 of calomel contain the ſame quantity of mercury as 113 of corroſive ſublimate, = 79. Theſe 79, with 11,5 of acid, are equal to 90,5, and leave 9,5 for the quantity of oxygen contained in calomel. It would appear, from theſe experiments, that corroſive ſublimate contains 6,5 per cent. more acid, and but 2,8 per cent. more oxygen, than calomel. But this quantity of oxygen is combined with a much greater proportion of mercury; and forms an oxide of a very different different degree of oxidizement. For, 88,5 : 9,5 : : 100 : 10,7. Therefore we may eſtabliſh the following comparative table.

CALOMEL.

The oxide of mercury in calomel is compoſed of,

| | |
|---|---|
| Mercury — — | 89,3 |
| Oxygen — — | 10,7 |
| | 100,0. |

And calomel is compoſed of,

| | | |
|---|---|---|
| Mercury 79 | oxide of | 88,5 |
| Oxygen 9,5 | mercury | |
| Muriatic acid — | | 11,5 |
| | | 100,0. |

CORROSIVE SUBLIMATE.

The oxide of mercury in corroſive ſublimate is compoſed of,

| | |
|---|---|
| Mercury — — | 85 |
| Oxygen — — | 15 |
| | 100. |

And corroſive ſublimate is compoſed of,

| | | |
|---|---|---|
| Mercury 69,7 | oxide of | 82 |
| Oxygen 12,3 | mercury | |
| Muriatic acid — | | 18 |
| | | 100. |

Theſe proportions are different from thoſe given by Lemery, Geoffroy, Bergman, &c. But, without calling in queſtion the accuracy and ſkill of theſe chemiſts, it is fair to aſſert, that the pure materials uſed by modern chemiſts, are more likely to lead to ſure reſults, than the impure reagents of the ancients.

In theſe ſalts we find another inſtance, that, in proportion as metallic oxides contain a greater quantity of oxygen, they require a greater quantity of acid to enter into combination with them.

The method I have followed, to aſcertain the proportions juſt ſtated, may appear, at firſt view, not to be the ſhorteſt that I might have adopted. But I have tried others, and have found none ſo accurate. It is impoſſible, ſynthetically, to convert a given quantity of mercury into calomel, in ſuch a manner as to be certain that none of it is in a different ſtate from that required. And, if we would attack calomel analytically, the action of the alkalis, without which we cannot proceed, is ſuch as to alter the nature of the oxides. I have alſo made many comparative experiments, by diſſolving calomel in nitro-muriatic acid, (which converted it into corroſive ſublimate) and then precipitating by ammonia; but I have not found theſe trials ſo ſuccesſful as thoſe I have deſcribed. The nature of the precipitate from corroſive ſublimate by ammonia, certainly differs, according to the exceſs of acid that may be preſent; and mercury ſeems to have the power of exiſting in many degrees of combination with oxygen. The only precaution abſolutely neceſſary in this mode of operating, is, that while the mercurial ſalt is in an open veſſel, it ſhould not be expoſed to a degree of heat capable of volatilizing any part of it.

The

The quantity of mercury ordered in the London Pharmacopoeia, to convert corrosive sublimate into calomel, is 9 pounds of mercury for every 12 pounds of corrosive sublimate. But, from the above experiments, it would appear, that a smaller quantity of mercury might strictly answer. However, from the results of minute investigation, we should not conclude too hastily upon preparations on the great scale; and I rather think, that the excess of mercury ordered by the Pharmacopoeia is a useful precaution.

In my experiments, I attempted to reduce, by means of copper, iron, or zinc, the mercury contained in the mercurial salts. Iron did not answer the purpose; zinc precipitated the mercury a little better; and copper produced a change which I did not expect. If a bit of copper be put into a solution of corrosive sublimate, a white powder shortly falls to the bottom; and that powder is calomel. When washed, it does not contain an atom of copper, nor of corrosive sublimate.

Before I conclude these considerations, I must say, that whether calomel be prepared in the dry or in the humid way,* it does not seem to differ chemically; nor does it contain any sensible

---

* By the humid way, I do not mean precisely the method of Scheele. That chemist desires us to boil the acid with the mercury, after they have ceased to act upon each other at a low temperature. By this method, the nitric acid takes up an excess of mercurial oxide; and the nitrate of mercury thus formed, precipitates by water. Therefore, when this nitrate of mercury is poured into the dilute solution of muriate of soda, according to the formula of Scheele, the action, on the part of the solution, is twofold.

1st. The water acts upon one part, and precipitates an oxide, or rather an insoluble subnitrate of mercury. And,

2dly, A double decomposition takes place between the nitrate of mercury and the muriate of soda. It is with reason that the medical world have supposed the calomel of Scheele to be different from that prepared in the humid way; for it is, in fact, calomel, plus an insoluble subnitrate of mercury. In the first part of Scheele's process, there is disengagement of nitrous gas, together with oxidizement and solution of some of the mercury. When he boils the acid upon the remaining mercury, there is no further disengagement of gas; yet more mercury is dissolved. The nitrate of mercury, therefore, rather contains an oxide less oxidized after ebullition than before it. The true difference is in the subnitrate of mercury, precipitated, as I before said, by the water in which the muriate of soda was dissolved. And the orange coloured powder, which remains after an attempt to sublime Scheele's calomel, is to be attributed to the same cause. To prepare calomel in the humid way, uniform as to itself, and in all respects similar to that prepared in the dry way, it is necessary, either to use the nitric solution before it is boiled, or to pour some muriatic acid into the solution of muriate of soda, previously to mixing it with the boiled solution of nitrate of mercury. In the first case, no precaution is necessary; and, in the latter, the oxide of mercury, which the nitrate of mercury has, by boiling, taken up in excess, finds an acid which fenfible portion of water of cryftallization. The fame may be faid of corrofive fublimate.

It now remains to fpeak of the real hyperoxygenized muriate of mercury. I paffed a current of oxygenized muriatic acid gas through fome water, in which there was red oxide of mercury. † After a fhort time, the oxide became of a very dark brown colour; and a folution appeared to have taken place. The current was continued for fome time; and when I thought that a fufficient quantity of the oxide had been diffolved, I ftopped the operation. The liquor was evaporated to drynefs; and the falt was thus obtained. There evidently was in the mafs a great proportion of corrofive fublimate, as might be expected, from what I had obferved to take place in the formation of the other falts of this acid; but, by carefully feparating the laft formed cryftals, I could pick out fome hyperoxygenized muriate of mercury. I then cryftallized it over again; and, in this manner, I obtained it nearly pure. This falt is more foluble than corrofive fublimate: about four parts of water retain it in folution. The fhape of its cryftals, I cannot well determine. When fulphuric, or even weaker acids, are poured upon it, it gives out the ufual fmell of hyperoxygenized muriatic acid; and the liquor becomes of an orange colour. This is a fufficient proof, that corrofive fublimate is not a hyperoxygenized muriate of mercury.

I have juft mentioned, that in the formation of this falt, the oxide of mercury, which was not diffolved by the acid, became of a very dark brown colour. I procured a portion of this oxide, which feemed different from the red oxide. It however retained the form, and the cryftalline appearance, of the latter. It was foluble in nitric acid, without difengagement of gas; and was precipitated from it, in a yellow oxide, by all the alkalis, except ammonia. It formed corrofive fublimate with muriatic acid; and the precipitate by the alkalis was the fame as that from corrofive fublimate, made with the red oxide. Yet I am inclined to think, that the dark brown oxide differs in fome effential point from the red; but I have not yet made fufficient experiments to prove this opinion. At all events, the prefent object being to examine the mercurial oxides only as combined with muriatic acid, it would be foreign to the purpofe,

---

which is ready to faturate it. All the mercurial oxide being thus converted into calomel, none of that fubnitrate of mercury can be prefent.

The objections made by a Medical gentleman againft Scheele's calomel, when this Paper was read before the Royal Society, led me to reconfider the fubject, and to undertake the inveftigation detailed in this Note.

† I ufed either of the red oxides of mercury, indifcriminately.

poſe, to enter upon too minute an inveſtigation of the other ſtates of the metal. This, and ſome other objects hinted at in this Paper, muſt be reſerved for future inquiry.

